# Observed mechanisms activating the recent subpolar North Atlantic Warming since 2016

**DOI:** 10.1098/rsta.2022.0183

**Published:** 2023-12-11

**Authors:** Léon Chafik, N. Penny Holliday, Sheldon Bacon, Jonathan A. Baker, Damien Desbruyères, Eleanor Frajka-Williams, Laura C. Jackson

**Affiliations:** ^1^ Department of Meteorology, Stockholm University, Stockholm, Sweden; ^2^ National Oceanography Centre, Southampton, UK; ^3^ Met Office, Hadley Centre, Exeter, UK; ^4^ Laboratoire d’Ocèanographie Physique et Spatiale (LOPS), Univ Brest, CNRS, Ifremer, IRD, IUEM, 29280 Plouzané, France; ^5^ University of Hamburg, Hamburg, Germany

**Keywords:** Atlantic meridional overturning circulation

## Abstract

The overturning circulation of the subpolar North Atlantic (SPNA) plays a fundamental role in Earth’s climate variability and change. Here, we show from observations that the recent warming period since about 2016 in the eastern SPNA involves increased western boundary density at the intergyre boundary, likely due to enhanced buoyancy forcing as a response to the strong increase in the North Atlantic Oscillation since the early 2010s. As these deep positive density anomalies spread southward along the western boundary, they enhance the North Atlantic Current and associated meridional heat transport at the intergyre region, leading to increased influx of subtropical heat into the eastern SPNA. Based on the timing of this chain of events, we conclude that this recent warming phase since about 2016 is primarily associated with this observed mechanism of changes in deep western boundary density, an essential element in these interactions.

This article is part of a discussion meeting issue ‘Atlantic overturning: new observations and challenges’.

## 1. Introduction

**Figure 1.**
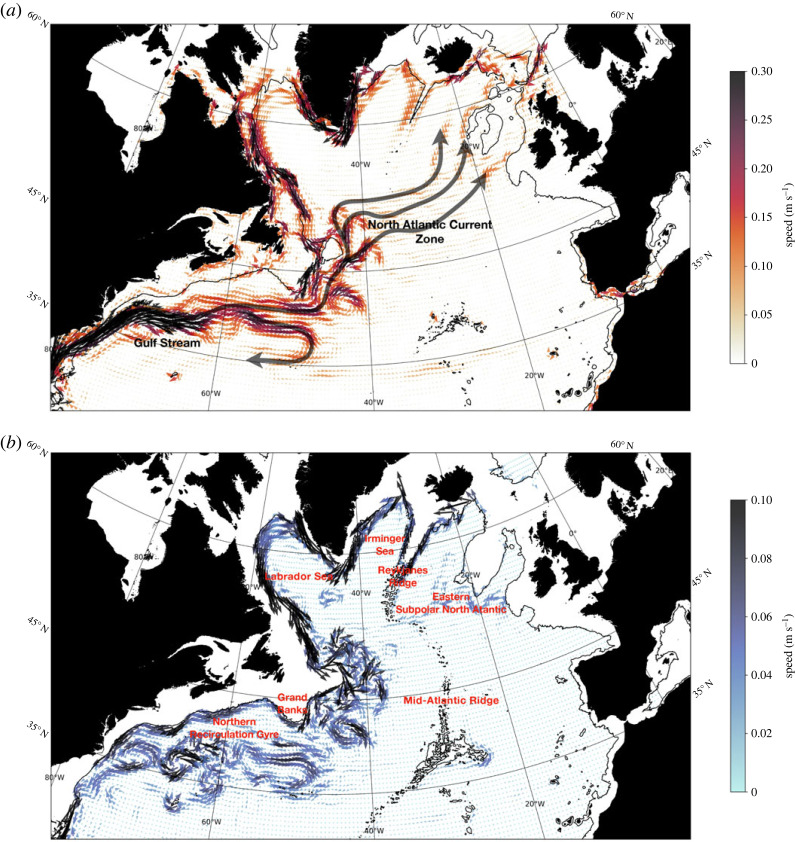
(*a*) The time-mean upper-ocean currents (0–1000 m) from the GloSea5 ocean reanalysis [5]. The vectors and colours indicate the direction and magnitude (speed) of the flow. The black text denotes the strongest upper-ocean currents in the North Atlantic. (*b*) Same as (*a*) but for the Deep Western Boundary Current path and its interior pathways as averaged in the 1400–2300 m layer. The red text is placed near the locations of the regions mentioned in the main text.

The North Atlantic Ocean (NAO) plays a fundamental role in Earth’s climate system due to the presence of the overturning circulation: warm and saline surface waters flow northward, become denser due to heat loss, sink at higher latitudes and return at depth towards the equator [1–4], figure 1. One of the key regions for the variability of the Atlantic meridional overturning circulation (AMOC) and hence for the northward oceanic heat transport is the subpolar North Atlantic (SPNA) [4,6], the circulation of which is thought to have played a significant role for the European climate over the last three millennia on centennial scales [7]. Furthermore, on decadal-to-multi-decadal scales, SPNA temperature changes, driven predominantly by AMOC variability and related northward heat transport, have been linked to important climate impacts such as Atlantic hurricane activity, Sahel rainfall and the climate of European and North American summers [8–11].

The SPNA circulation and concomitant thermohaline changes, especially its rapid warming and salinification in the mid-1990s, have been dealt with in numerous studies [12–16] and important insights regarding the mechanisms have been gained from this decadal event (see also [3,4]). The modelling work among these have generally connected the observed increase of warm and saline subtropical waters into the SPNA in the mid-1990s to a strengthened state of the AMOC (the maximum streamfunction in latitude-depth space) in response to a prolonged positive NAO in the early 1990s. Robson *et al.* [15] further suggested on the basis of model experiments that the primary cause of the mid-1990s SPNA warming is mainly due to increased ocean heat transport forced by enhanced buoyancy loss (induced by persistently strong NAO period) and less so due to changes in wind stress forcing. This model-based conclusion disagrees to some extent with the study of Häkkinen *et al.* [17] where spatial shifts of the zero wind-stress curl are suggested to regulate the northward access of subtropical waters into the SPNA (see also [18]).

Since around 2016, and following the cold SPNA period that lasted for nearly a decade [18–24], including intense heat loss events between 2013 and 2015 that induced the North Atlantic cold blob [25], a shift in ocean circulation is reported to have initiated a new SPNA warming phase [26]. The findings from the latter study indicate that this warming is caused by enhanced advection of subtropical waters to the eastern SPNA (region east of the Reykjanes Ridge, figure 1*b*), and to some extent by a reduction in surface net heat loss. This is consistent with Desbruyères *et al.* [27] where an AMOC-induced warming of the SPNA since 2016 was statistically predicted based on the surface-forced watermass transformation framework, which implicitly includes the gyre circulation, as further elaborated on in the methods section. The mechanisms that activated this enhanced influx of subtropical heat into the SPNA remain poorly understood, hence providing the motivation for this work.

One of the mechanisms we will be focusing on involves the southward progression of density anomalies originating from the western SPNA, which here include the Irminger and Labrador Seas (figure 1). These anomalies have been suggested to be closely connected to the strength of the Atlantic overturning and related northward heat transport [19,20,28,29]. For instance, Williams *et al.* [29] conducted a semidiagnostic dynamical model analysis of historical hydrographic data and concluded that subpolar heat content changes are primarily driven by ocean heat convergence and altered via changes in depth-integrated western boundary density that communicate southward from the Labrador Sea. In another study, Jackson *et al.* [20] reported that negative deep subpolar density anomalies in the GloSea5 ocean reanalysis from 2003 to 2010 communicated with the subtropics in 2008–2015. This negative decadal density change was found to be consistent with changes in the subtropical AMOC and its associated heat transport, potentially contributing to the observed cooling experienced in the eastern SPNA since the mid-2000s [19,23]. The degree to which southward communication of deep density anomalies originating from the western SPNA is involved in driving the recent warming observed in the eastern SPNA will be investigated in the present study.

The structure of the study is as follows: We begin by gaining insights into the spatio-temporal variations of relevant ocean climate variables. We then examine the evolution of deep western boundary density anomalies and how they relate to NAO forcing as well as changes in the North Atlantic Current (NAC) through sea-surface gradient at the intergyre boundary. Following this, we present the temporal variability of meridional ocean heat transport estimates and its components from a set of ocean reanalyses with the aim to establish a connection to deep western boundary densities, atmospheric forcing and basin-scale sea-surface height changes to elucidate the observed patterns during the recent warming period. A conclusion section ends the paper.

## 2. Data and methods

### (a) Satellite-based sea surface heights

We use the global monthly absolute dynamic topography, which refers to the sea-surface height above geoid, derived from satellite altimetry and retrieved from the AVISO (Archiving, Validation and Interpretation of Satellite Oceanographic data) FTP server. The global maps are based on the delayed-time ‘allsat’ product, i.e. the merged maps computed with all the satellites available at a given time. The grid resolution is $0.25^{\circ }\times 0.25^{\circ }$ and the data spans the 1993–2021 period. The sea-surface height anomalies used in the study have been constructed by first removing the seasonal cycle and linearly detrending the data over the 1993–2021 period.

### (b) Hydrography

We use the Chinese Institute of Atmospheric Physics (IAP) global ocean temperature and salinity products, which have a grid resolution of $1^{\circ }\times 1^{\circ }$ and include 41 vertical standard depth levels ranging from 0 to 2000 m, with monthly temporal resolution from 1993 to 2021. To ensure data quality, the IAP hydrographic data were bias-corrected for depth error, temperature error and probe type using XBT measurements and other *in situ* observations retrieved from the World Ocean Database. The mapping of temperature and salinity is based on the dynamical ensemble approach, as detailed in Cheng & Zhu [30]. This assimilation technique uses an Ensemble Optimum Interpolation method combined with covariance from CMIP5 multi-model simulations. This approach takes advantage of the capability of these models to simulate the general ocean circulation, enabling a more accurate estimation of the covariance. The method is particularly advantageous for reconstructing historical ocean subsurface variations. Additionally, we used the IAP gridded global ocean heat content and steric height (0–2000 m) products, which also have a grid resolution of $1^{\circ }\times 1^{\circ }$.

The deep density anomalies in this study are computed based on the average densities in the 1400–2000 m layer using TEOS-10 equation of state [31]. The classical Labrador Sea Water is typically found within the depths of 1400–2300 m [32]. However, due to the limitations of the available IAP data, which only extend down to 2000 m, we are constrained to using the 1400–2000 m layer for our analysis. This is still a valid approach, as previous work [20] has shown that a single layer at a depth of 1795 m is sufficient to capture the propagation of deep density anomalies from the subpolar region to the subtropics. The anomalies of the IAP data have been constructed by first removing the seasonal cycle and linearly detrending the data over the 1993–2021 period.

### (c) Meridional ocean heat transport from ocean reanalyses

We calculate the total meridional ocean heat transport (MHT) at the intergyre region using the following formulation in depth coordinates [33]:

2.1 $$\mathrm{MHT}=\rho c_{p}\int_{-D}^{0}\int_{x_{w}}^{x_{e}}V\;\theta \;dx\;dz,$$

where $C_{p}$ is specific heat capacity ($3994\;{J\;\mathrm{kg}}^{-1}\;C^{-1}$), ρ is density of seawater ($1025\;{\mathrm{kg}\;m}^{-3}$), V is meridional velocity (corrected to ensure net zero meridional volume transport) and θ is potential temperature. The integration is done over the entire water column (*D*) and stretches from the eastern ($x_{e}$) to the western boundary ($x_{w}$). Following Hall & Bryden [33], we further partition the MHT into the vertical (${\mathrm{MHT}}_{\mathrm{vert}}$) and the horizontal gyre (${\mathrm{MHT}}_{\mathrm{gyre}}$) components of the heat transport using the following expressions:

2.2 $${\mathrm{MHT}}_{\mathrm{vert}}=\rho c_{p}\int_{-D}^{0}L(z)\bar{V}\;\bar{\theta }\;dz$$

and

2.3 $${\mathrm{MHT}}_{\mathrm{gyre}}=\rho c_{p}\int_{-D}^{0}\int_{x_{w}}^{x_{e}}V^{\prime }\;\theta^{\prime }\;dx\;dz,$$

where the overbars in ${\mathrm{MHT}}_{\mathrm{vert}}$ indicate zonal averages and L(z) is the corresponding ocean basin width at a given depth. The primes in ${\mathrm{MHT}}_{\mathrm{gyre}}$ indicate deviation from the zonal average. This distinction helps to clarify the respective contributions of the vertical or overturning and horizontal gyre circulation to the overall MHT at the intergyre boundary.

The MHT decomposition at the intergyre region includes components driven by the circulation associated with transformation of water from shallow to deep layers (referred to as ‘vertical’ circulation or overturning in depth space) and the circulation associated with the subpolar gyre (referred to as ‘horizontal’ or gyre circulation in depth space). The purpose of this approach in the present study is to elucidate the mechanisms that control the vertical and horizontal MHT variability, which are not likely to be the same processes [34]. This choice may seem counterintuitive given recent emphasis (following Lozier *et al.* [35]) on the computation of AMOC in density space at the OSNAP array, so we explain further. The transformation of lighter water to denser water in the SPNA is a gradual process with the upper layer steadily losing buoyancy as it circulates along pathways around the gyre (figure 1), leading to isopycnals that slope from east to west [35,36]. This means that, geographically, the subpolar gyre is an integral part of the AMOC pathway and the subpolar AMOC computed in density space is a rate of transformation from light to dense, shallow to deep, and east to west, and includes the gyre. If our goal is to estimate the total diapycnal transformation from light water to dense, the subpolar AMOC in density space provides that and is larger than that computed in depth space. However, while total MHT computed in density space and depth space are by definition identical, in order to examine the importance of the gyre in MHT, the decomposition needs to be in depth space.

The MHT calculations are performed using the monthly mean fields (1993–2020) of the global eddy-resolving ocean reanalysis, GLORYS12, retrieved from the Copernicus Marine Environment Monitoring Service (CMEMS, https://data.marine.copernicus.eu/product/GLOBAL_MULTIYEAR_PHY_001_030/description). The ocean model component of GLORYS12 is the NEMO platform driven by ERA-interim atmospheric forcing until 2018 and ERA5 thereafter [37]. GLORYS12 has a horizontal resolution of $1/12^{\circ }$ and 50 vertical levels. For the MHT calculations, we use the latitudinally averaged ($45^{\circ }\;\text{N--}46^{\circ }\;N$) meridional velocities and potential temperatures stretching from the western to the eastern boundary. We also take advantage of the sea-surface heights in the wider North Atlantic from GLORYS12 to examine their relationship to the MHT variability. Furthermore, we evaluate the GLORYS12 MHT calculations using the ensemble mean and spread of four ocean reanalyses (GLORYS2V4, ORAS5, GloSea5 and C-GLORSv7), referred to as ${\mathrm{MHT}}_{\mathrm{grepv}2}$. These ocean reanalyses have a horizontal resolution of $1/4^{\circ }$ and 75 vertical levels, and have been shown to provide reasonable estimates of the overturning and MHT when compared with OSNAP observations [38]. They are also available through CMEMS (https://data.marine.copernicus.eu/product/GLOBAL_REANALYSIS_PHY_001_031/description).

### (d) The leading mode of atmospheric variability

We use the monthly zonal and meridional wind stress components from the NCEP/NCAR reanalysis [39] to calculate the wind stress curl over the 1948–2019 period. The grid resolution of the data is $2.5^{\circ }\times 2.5^{\circ }$. Based on the deseasoned and detrended monthly wind-stress curl fields, we calculate the leading Empirical Orthogonal Function mode [40] of wind-stress curl variability over the NAO for the entire period (1948–2019). The spatial mode of this variability mainly reflects the NAO [41], with positive wind-stress curl anomalies located south of Iceland in the Irminger Sea, and negative anomalies in the subtropical region near the Azores, and explains 23.2% of the variance.

## 3. Results and discussion

### (a) Insights into the evolution of the recent warming

**Figure 2.**
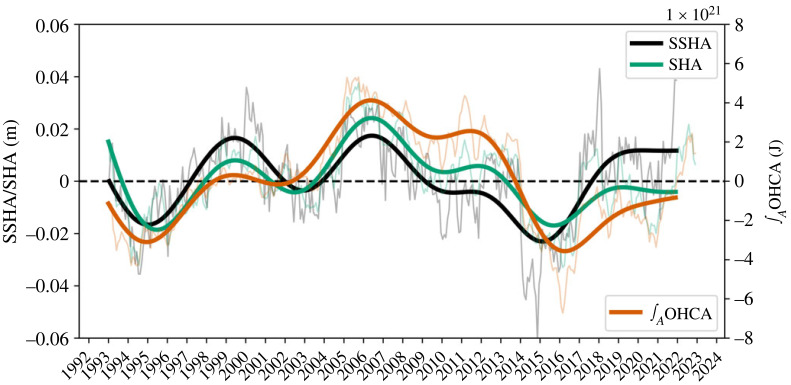
Time series of the averaged sea-surface height anomaly (SSHA, m), averaged steric height anomaly (SHA, m, 0–2000 m) and area-integrated ocean heat content anomaly (OHCA, J, 0–2000 m) in the eastern SPNA (see dashed box in figure 3*c*). The anomalies are constructed by removing the seasonal cycle and the 1993–2021 linear trend. The thick lines are constructed by applying a fourth-order Butterworth filter with a 36-month cut-off window on the monthly fluctuations (thin lines) to represent interannual-to-decadal variability.

**Figure 3.**
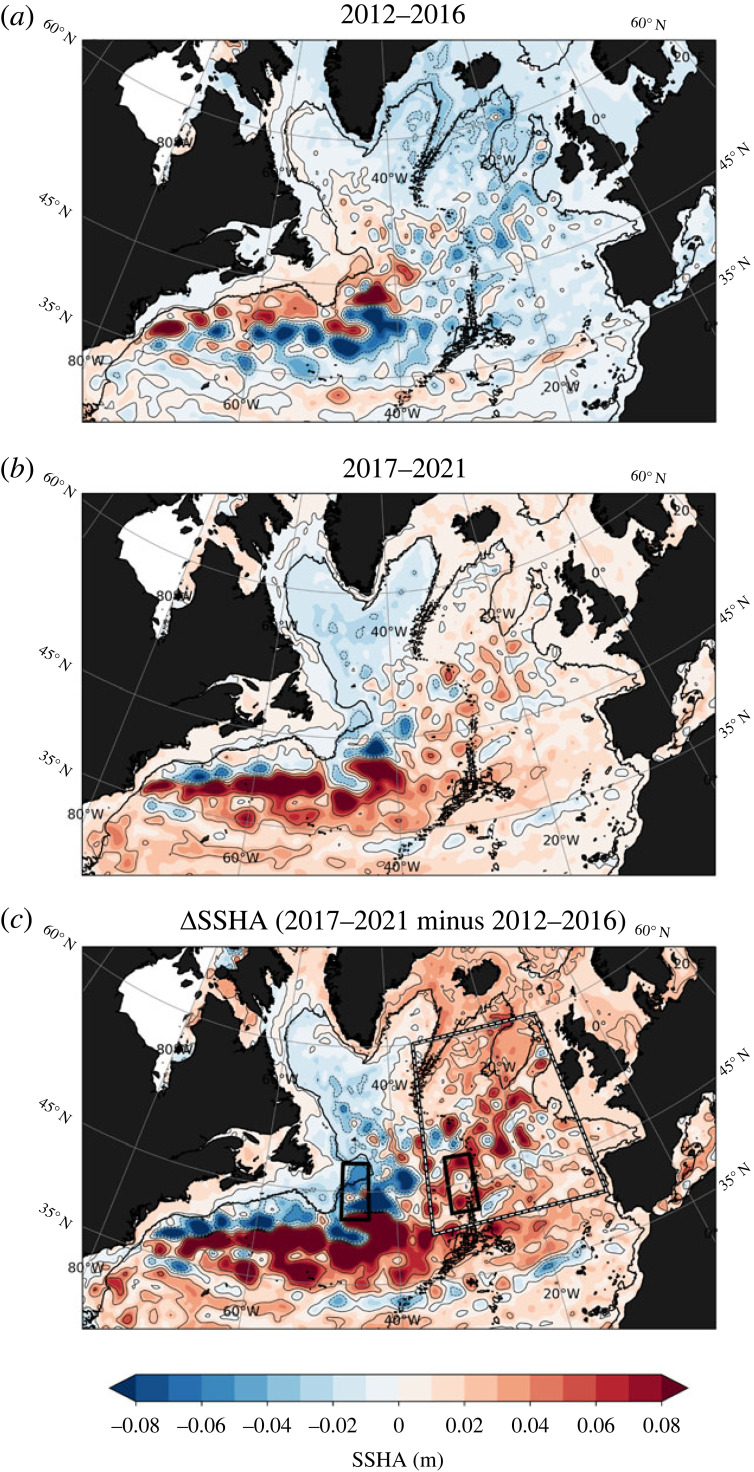
(*a*) The 2012–2016 average of North Atlantic SSHA (m). (*b*) Same as (*a*) but for the 2017–2021 period. (*c*) The SSHA difference between the two periods shown in (*a*) and (*b*). The anomalies are constructed by removing the seasonal cycle and the 1993–2021 linear trend before calculating the difference between the two periods. The black contour in all panels depicts the 1795 m isobath.

We commence by examining the evolution of satellite-derived sea-surface height anomalies, steric height anomalies and ocean heat content anomalies in the eastern SPNA (see dashed box in figure 3*c*), as illustrated in figure 2. These climate variables are strongly intercorrelated and collectively reveal the dominant decadal variability in the SPNA. Notably, the decadal warming during the 1996–2005 period is clearly evident, followed by a decadal cooling trend until the mid-2010s. The impact of extreme winters in the SPNA between 2013 and 2015 is evident as a sharp drop in these variables before a subsequent upturn around 2016 is observed, which marks the onset of the new warming phase [26].

The progression shown in figure 2 also demonstrates that the tendency in sea-surface heights over the eastern SPNA is predominantly of steric origin, suggesting that the recent thermal reversal in this region is likely driven by advective processes, which is in agreement with the conclusion of Desbruyères *et al.* [26]. By decomposing the eastern SPNA into its main source waters (subtropical and subpolar) within the upper ocean, they were able to reconstruct and quantify the changing relative proportion of subtropical and subpolar waters using an altimetry-based advective model and a statistical clustering of ocean *in situ* profiles. Their analysis revealed an increase in the volume of subtropical waters since around 2016, which was attributed to shifts in the horizontal structure of the large-scale upper ocean circulation. We here argue that these changes in the horizontal circulation and steric sea-surface heights are indicative of increased northward heat transport across the intergyre boundary, primarily driven by southward propagation of deep density anomalies of subpolar origin along the western boundary, as will be shown later in the text.

We now explore the spatial sea-surface height anomaly pattern associated with part of the decadal cooling period that dominated the waters east of Greenland in the SPNA, figure 3*a*. The negative sea-surface height anomaly observed during this 5-year period (2012–2016) coincided with enhanced watermass transformation and overturning in density space as estimated from the OSNAP East array [4,27,42]. These observations [35] revealed that watermass transformation associated with the maximum overturning value in the SPNA occurs primarily between Greenland and Scotland in the Iceland Basin and Irminger Sea driven by local buoyancy forcing [42]. By contrast, minimal change in sea-surface height anomalies was observed in the Labrador Sea during this 2012–2016 period. At the intergyre boundary (approx. $45^{\circ }\;N$), an anomalous sea-surface height increase and decrease near the Grand Banks of Newfoundland and in the vicinity of the mid-Atlantic Ridge, respectively, is visible (figure 3*c*). This anomalous negative zonal sea-surface height gradient at the intergyre region, which also coincides with a northward-shifted Gulf Stream position, induces a relatively weak northward flow geostrophic flow, which probably relates to decreased ocean heat supply by the NAC to the eastern SPNA during the 2012–2016 period. It should, however, be noted that the extreme winters between 2013 and 2015 [25] have strongly contributed to the rapidity of the cooling and decrease in sea-surface heights, amplifying the internal variability of the system (as evident from the sharp decrease in figure 2).

The sea-surface height anomaly map highlighting the 2017–2021 period shows, however, an indication that the low sea-surface height anomaly that dominated the eastern SPNA during the 2012–2016 period has propagated westwards, figure 3*b*. This westward propagation of sea-surface height anomalies is thought to be linked to shifting wind patterns and to the cyclonic time-mean ocean circulation in the SPNA, as shown recently by Volkov *et al.* [43]. The eastern and western boundary are now experiencing anomalous sea-surface height increase and decrease, respectively, that is particularly enhanced at the intergyre boundary, as visible from the difference between the two periods (figure 3*c*). As a result of this anomalous positive zonal sea-surface height gradient at the intergyre boundary, an enhanced northward geostrophic flow by the NAC is anticipated, which would in turn increase the supply of subtropical heat in the eastern SPNA. Note that during this recent warming period, there has been a concurrent southward migration of the Gulf Stream, likely as a result of changing western boundary density.

**Figure 4.**
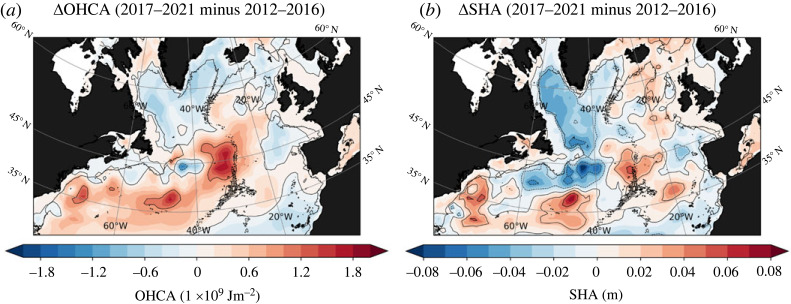
(*a*) Ocean heat content anomaly (OHCA) difference between the two 5-year periods (2012–2016 and 2017–2021). (*b*) Same as (*a*) but for steric height anomaly (SHA). The anomalies are constructed by removing the seasonal cycle and the 1993–2021 linear trend before calculating the difference between the two periods. The black contour on the maps depicts the 1795 m isobath.

The difference in the integrated ocean heat content anomalies (0–2000 m) between the two 5-year periods (2012–2016 and 2017–2021) reveals evidence of increased heat content in the subtropics, extending northeastward towards the mid-Atlantic Ridge and crossing the intergyre boundary into the eastern SPNA, figure 4*a*. This anomalous change in heat content along this pathway is also reflected in the integrated steric height anomalies (0–2000 m), figure 4*b*. However, along the western boundary of the subpolar gyre and at the intergyre boundary, the difference in the integrated steric height anomalies between the two periods is largely negative. This steric height anomaly pattern aligns with that of sea-surface heights (cf. figure 3, although the 0–2000 m steric height signal is, as expected, more pronounced), both revealing of an anomalously strong west-east gradient at the intergyre boundary (more specifically between the Grand Banks of Newfoundland and in the vicinity of the mid-Atlantic Ridge), supporting the notion of anomalous surface heat advection into the eastern SPNA by the NAC.

### (b) Southward spreading of deep density anomalies from hydrography

**Figure 5.**
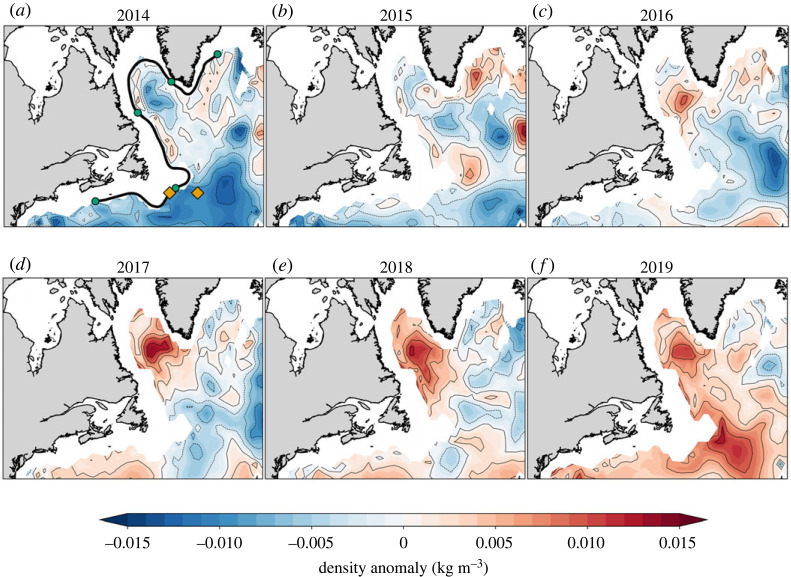
(*a*–*f*) Evolution of North Atlantic deep density anomalies (${\mathrm{kg}\;m}^{-3}$) between 2014 and 2019 in the western boundary. The deep density anomalies have been constructed by averaging density in the 1400–2000 m layer, removing the seasonal cycle, and detrending the data for the 1993–2021 period. The black contour in *a* is the 1795 m isobath representing the DWBC used in figure 6*b*. The orange diamonds in the same panel denote the site of the DWBC and interior pathways at the intergyre boundary (at $45^{\circ }\;N$) used in figure 7.

In this section, we examine the spatio-temporal variability of deep density anomalies along the Deep Western Boundary Current (DWBC) path, figure 5*a*. The southward spreading of deep positive density anomalies along the DWBC and its interior pathways is expected via thermal wind-balance to enhance the southward geostrophic transport. The conservation of mass dictates that there must be a compensating flow in the opposite direction to maintain mass continuity. This implies that there will be an increased northward upper ocean flow, mainly by the NAC, in response to the increased deep southward geostrophic flow.

**Figure 6.**
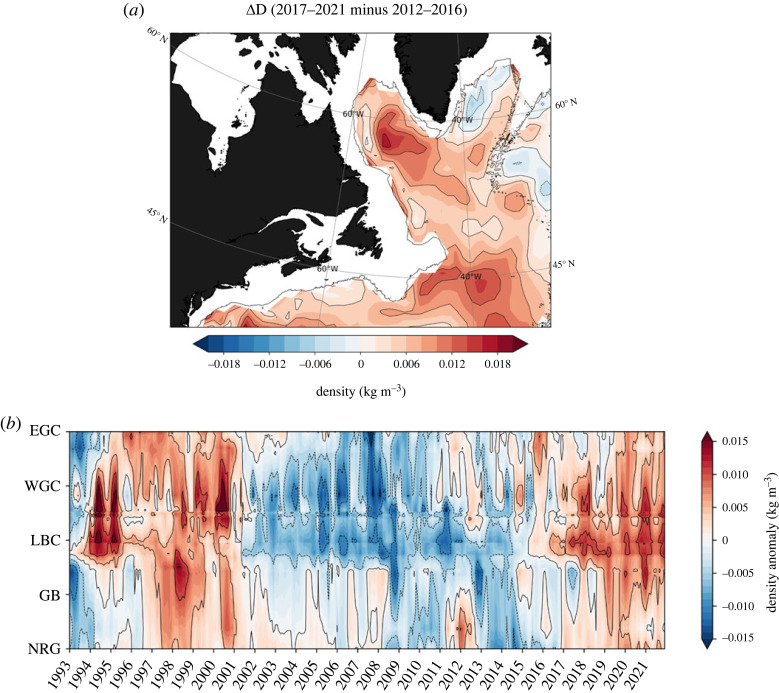
(*a*) Deep density anomaly difference (ΔD) between the two 5-year periods (2012–2016 and 2017–2021). The black contour depicts the 1795 m isobath. (*b*) Hovmöller diagram of observed deep density anomalies averaged in the 1400–2000 m layer. These monthly unfiltered deep density anomalies have been constructed by averaging density in the 1400–2000 m layer, removing the seasonal cycle, and detrending the data for the 1993–2021 period. The diagram traces the 1795 m isobath (cf. black contour and green circles in figure 5*a*) connecting the East Greenland Current (EGC), the West Greenland Current (WGC), the Labrador Current (LC), the Grand Banks (GB) and the northern wall of the Northern Recirculation Gyre (NRG).

The spatial progression of deep positive density anomalies for the 2014–2019 period from hydrographic observations is presented in figure 5. In essence, there is a progressive increase of deep positive density anomalies along both the western boundary current and the interior pathways during this 6-year period [44–46]. Of particular significance is the accumulation of dense water in the central Labrador Sea since approximately 2016–2017, followed by its clear ejection into the interior near the Grand Banks in the vicinity of the intergyre boundary around 2018–2019. The difference in the deep density anomalies between the two 5-year periods (2012–2016 and 2017–2021) reveals a notable basin-wide increase and persistent positive anomalies along the entire western boundary (figure 6*a*). The increased influx of heat into the eastern SPNA in recent years by the NAC, in particular post-2016, can thus be related to the progressive increase in the deep density anomalies along the western boundary. This is consistent with Williams *et al.* [29], which used historical hydrographic data assimilated into a dynamical model to estimate the heat convergences and concluded that the decadal subpolar warming and cooling trends are linked to changes in the depth-integrated Labrador Sea density (similar to the steric height anomaly change, figure 4*b*). A similar conclusion was recently drawn by Yeager *et al.* [47], which further emphasized, based on a multi-century preindustrial climate model simulation, the essential role of deep dense anomalies of Labrador Sea origin in the multi-decadal ocean memory of the NAO (see also reference [48]).

A careful examination of the observed southward communication of deep density anomalies along the DWBC from hydrography further reveals a noticeable low-frequency variability in the subpolar gyre (figure 6*b*). This observed temporal variability of the deep density anomalies are broadly consistent with the decadal subpolar AMOC change, which is a rate of watermass transformation and includes the horizontal gyre circulation, between Greenland and Scotland from GloSea5 [4,49] as well as the tendency in ocean heat content in the eastern SPNA (figure 2). The Hovmöller diagram further reveals a southward communication of the deep positive density anomalies along the DWBC in the recent years concomitant with the increase in ocean heat content in the eastern SPNA. This southward communication bears similarities to the mid-1990s warming event [15]. Additionally, there is an indication that negative deep density anomalies in the mid-2000s in the subpolar gyre communicated southward with the subtropics. This is consistent with the results of Jackson *et al.* [20], which reported using the GloSea5 ocean reanalysis that the propagation of subpolar-origin decadal density anomalies in the mid-2000s contributed to the reduction of the subtropical AMOC transport. The recent increase in deep density anomalies along the western boundary and subsequent ocean circulation changes may thus be a recovery from an earlier decrease. However, it is worth noting that this increase may have been amplified by the strong buoyancy forcing driven by extreme winter conditions in 2013–2015, which led to intense watermass transformation in the SPNA [25,27,42,50].

### (c) Deep western boundary density and relationship to NAC strength

**Figure 7.**
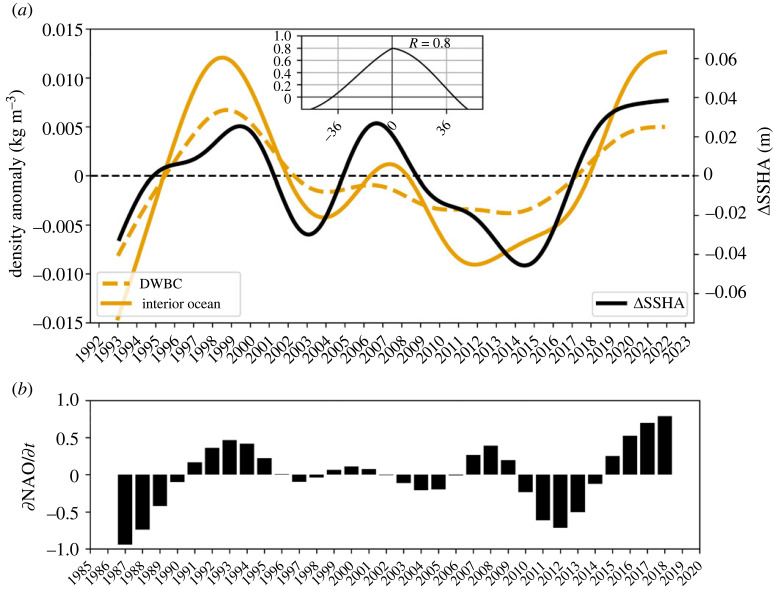
(*a*) Deep density anomalies at the DWBC (dashed orange) and interior ocean (orange) sites at the intergyre boundary (cf. figure 5*a*). The black line shows the gradient in sea-surface height anomaly taken between the western boundary and near the mid-Atlantic Ridge (see black boxes in figure 3*c*). The times series have been smoothed using a fourth-order Butterworth filter. The inset represents the cross-correlation between the smoothed deep density anomalies (an average of the two orange time series) and the smoothed ΔSSH at the intergyre boundary (black line). The corresponding correlation coefficient, *R* = 0.8 (significant at the 95% confidence level), at zero lag is shown within the inset. (*b*) The tendency in the NAO forcing (black bars), ∂NAO/∂t, between 1987 and 2019. The NAO index has also been smoothed with the same filter used in *a*.

**Figure 8.**
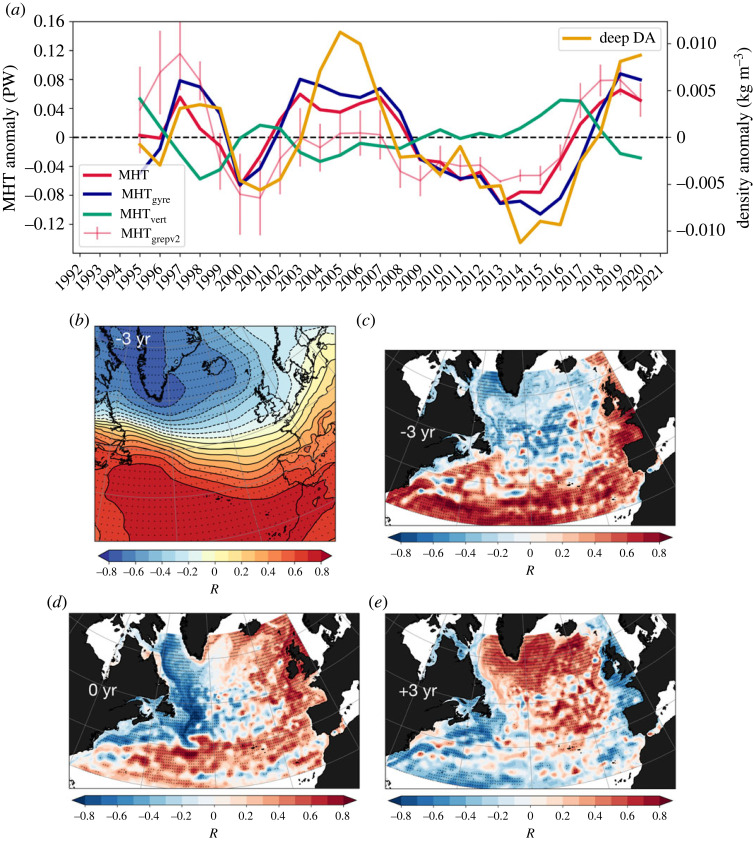
(*a*) Temporal variability (1993–2020) of MHT (red), ${\mathrm{MHT}}_{\mathrm{gyre}}$ (dark blue), MHT${\;}_{\mathrm{vert}}$ (green) anomaly estimated at $45^{\circ }$N from the $1/12^{\circ }$ GLORYS12 ocean reanalysis. Deep density anomalies at the 1684 m level and averaged at two sites (DWBC and interior ocean) at $45^{\circ }\;N$ (orange). For robustness, the ensemble mean temporal variability of MHT anomaly from the $1/4^{\circ }$ ocean reanalyses and associated ensemble spread are also included (thin pink). (*b*) Three-year lag-correlation pattern between MHT anomaly from GLORYS12 and sea-level pressure anomaly from ERA5 (1993–2020). (*c*) Same as (*b*) but for GLORYS12 SSHA. (*d*) Same as (*c*) but no lag applied (zero correlation). (*e*) Same as (*d*) but the pattern represents 3-year lead correlations. The anomalies have been constructed by removing the seasonal cycle, detrending the data for the 1993–2020 period and smoothing the annual mean anomalies using a 3-year running mean. The stipplings indicate significance at the 95% confidence level using a two-sided *t*-test.

The temporal variability of western boundary density, in particular at the intergyre region, exhibits a clear decadal pattern that characterizes the DWBC as well as interior pathways (figure 7*a*). It should, however, be acknowledged that density anomalies at both sites (figure 5) might not be entirely independent because of the effective resolution of the hydrographic data used here. This aspect should be recognized as a limitation of the presented density time series. The anomalous increase in densities associated with the warming of the mid-1990s was followed by a gradual decrease until the early 2010s, before a gradual upward trend in western boundary density at both sites became apparent after 2012, with a more rapid increase after 2016. This positive trend in density anomaly could potentially be linked to an increase in NAO forcing. This is indicated by the positive trend in NAO tendency since 2012 (figure 7*b*) and supported by the implied overturning in density space from observed buoyancy fluxes (cf. fig. 2*c* in [4]) as well as the notable increase in the vertical component of the MHT during this period as estimated from ocean reanalyses (figure 8*a*). As noted previously, the southward spreading of deep density anomalies and the increase in southward transport are accompanied by an increase in northward transport. The immediate response to the arrival of these buoyancy anomalies is a strengthening of the NAC and associated east-west sea-surface height gradient at the intergyre boundary (cf. figures 3–4). This explains the strong correlation at zero lag, R=0.8, between deep western boundary density (a combination of both the DWBC and interior ocean) and sea-surface height gradient at the intergyre boundary (inset in figure 7*a*). As such, the observed changes in sea-surface height gradient or NAC strength at the intergyre boundary on these timescales (figure 3) is a response to changes in the subpolar overturning circulation and associated southward spreading of deep density anomalies along the western boundary. These observational-based results are consistent with the modelling study of Yeager *et al.* [47], which provides a discussion along these lines on this mechanism but particularly highlights the significant driving role of anomalous production of deep Labrador Sea Water for the multi-decadal variability of northward heat transport at the intergyre boundary.

In the following section, we deconstruct the MHT at the intergyre boundary from ocean reanalyses, determine which component dominates the total MHT variability in this region and understand the linkages to atmospheric forcing and basin-scale sea-surface height anomaly patterns with the aim to enhance the robustness of the observed connections described so far.

### (d) Meridional ocean heat transport variability and linkages

To establish a robust connection between recent SPNA warming and changes in ocean circulation, we analyse the temporal variability of MHT anomaly and its components over the 1993–2020 period as seen by ocean reanalyses. Figure 8*a* presents evidence of a rapid increase in the total MHT since mid-2010s. Both the high-resolution ocean reanalysis, GLORYS12, and the ensemble mean of the $1/4^{\circ }$ set of ocean reanalyses corroborate this notable increase. While this surge in total MHT involves some elements of the MHT${\;}_{\mathrm{vert}}$, particularly between 2012 and 2016 (a period characterized by enhanced watermass transformation), it appears predominantly driven by the anomalous changes in MHT${\;}_{\mathrm{gyre}}$ at the intergyre boundary. This component accounts for a substantial 84% of the explained MHT variance, underscoring its critical role in shaping the pronounced interannual-to-decadal temperature fluctuations in the SPNA.

The temporal variability of the ${\mathrm{MHT}}_{\mathrm{gyre}}$ anomaly also shows a strong connection with deep western boundary density anomalies, i.e. accounting for both boundary and interior pathways at the western intergyre region. This relationship holds particular significance as it indicates that not only does the timing of the recent warming align with anomalous increase in ${\mathrm{MHT}}_{\mathrm{gyre}}$, it also corresponds with the arrival of basin-wide positive deep density anomalies at the intergyre boundary. This alignment implies that deep density anomalies of subpolar gyre origin, likely formed by enhanced watermass transformation, play an essential role in driving the influx of subtropical heat by the NAC into the eastern SPNA.

Our results further suggest that the anomalous increase in total MHT and deep density anomalies at the intergyre boundary are primarily driven by the NAO, which is found to precede these signals by about 3 years (figure 8*b*) and notably imprints on SSHA both encircling the rim of the subpolar gyre and over the subtropical gyre (figure 8*c*). The instantaneous correlation pattern between the MHT anomaly and SSHA (figure 8*d*) displays a distinct horse-shoe pattern characterized by large-scale SSHA gradients, i.e. decreased SSHA along the western boundary and increased SSHA at the eastern boundary (the corresponding upper-ocean temperature anomaly correlation pattern displays positive correlations tracing the NAC path into the eastern SPNA, not shown). It is noteworthy that this pattern closely mirrors the resultant SSHA pattern observed between the two 5-year periods (figure 3*c*).

This large-scale SSHA pattern, which is followed by a basin-scale warming of the SPNA (figure 8*e*), thus emerges on these interannual-to-decadal timescales as a result of increased MHT at the intergyre boundary driven mainly by the NAC (note the strong SSHA gradients) as a result of strengthened NAO forcing (primarily through buoyancy forcing) and southward communication of deep positive density anomalies of subpolar origin along the western boundary. It is thus reasonable to conclude that the major driver of changes in the horizontal circulation and associated MHT during this recent warming event is due to changes in the ocean density structure rather than wind stress curl forcing, consistent with earlier studies [51,52].

## 4. Conclusion

We have in this study examined the mechanisms activating the recent warming phase in the eastern SPNA since 2016, focusing specifically on the southward communication of deep density anomalies along the DWBC. Our results indicated that this warming event likely involved the prolonged positive NAO tendency since about 2012 and associated buoyancy forcing that led to increased watermass transformation [4,27,42,49]. We found observational evidence that deep western boundary density anomalies at the intergyre boundary started to increase following this buoyancy forcing mechanism. This timing aligned with the onset of the warming in the eastern SPNA caused by enhanced influx of subtropical heat by the NAC. The coupling between the lower and upper ocean transports can thus be ascribed to the southward propagation of deep density anomalies, which in turn strengthens the horizontal circulation and associated meridional heat transport.

Southward propagation of deep positive density anomalies along the western boundary is therefore considered to be an essential element for the development of this recent SPNA warming. Because of the multi-year chain of events that involved changes in the buoyancy field, their timing, and the associated NAC adjustment, it is reasonable to conclude that changes in surface wind stress curl forcing are less likely to have played a significant role in activating the upturn in heat content since 2016 in the eastern SPNA.

## Data Availability

Altimetry: https://www.aviso.altimetry.fr/en/data/data-access/ftp.html, hydrography: http://www.ocean.iap.ac.cn, wind: https://psl.noaa.gov/data/gridded/data.ncep.reanalysis.html, wind: https://cds.climate.copernicus.eu/cdsapp#!/dataset/reanalysis-era5-single-levels-monthlymeans?tab=overview, reanalyses: (1) https://data.marine.copernicus.eu/product/GLOBAL_MULTIYEAR_PHY_001_030/description (2) https://data.marine.copernicus.eu/product/GLOBAL_REANALYSIS_PHY_001_031/description.
